# Long‐Term Follow‐Up of Neoadjuvant Enzalutamide Plus Androgen Deprivation Therapy in Localized Prostate Cancer: A Secondary Analysis of a Neoadjuvant Feasibility Trial

**DOI:** 10.1002/pros.70093

**Published:** 2025-11-16

**Authors:** Braden Millan, Nikhil Pramod, Ruben Blachman‐Braun, Jaskirat Saini, Lauren Loebach, Milan Patel, Sandeep Gurram, Baris Turkbey, Fatima Karzai, Peter A. Pinto

**Affiliations:** ^1^ Urologic Oncology Branch, National Cancer Institute, National Institutes of Health Bethesda Maryland USA; ^2^ Molecular Imaging Branch, National Cancer Institute, National Institutes of Health Bethesda Maryland USA; ^3^ Genitourinary Malignancies Branch, National Cancer Institute Bethesda Maryland USA

**Keywords:** androgen receptor signaling inhibitors (ARSIs), cancer‐specific survival (CSS), neoadjuvant therapy, prostate cancer, robot‐assisted radical prostatectomy (RARP)

## Abstract

**Introduction:**

Neoadjuvant intense androgen deprivation therapy (ADT) with androgen receptor signaling inhibitors (ARSIs) has shown pathologic complete responses (pCR) in prostate cancer (PCa), but long‐term survival outcomes remain unclear. This study evaluates the durability of response following neoadjuvant ADT plus enzalutamide before robot‐assisted radical prostatectomy (RARP) and lymph node dissection.

**Methods:**

We conducted a secondary analysis of an open‐label feasibility trial enrolling men with NCCN intermediate‐, high‐, very high‐risk localized and regional PCa treated with 6 months of neoadjuvant ADT and enzalutamide. Factors associated with biochemical recurrence (BCR) and metastases were evaluated using appropriate univariable statistical tests, and BCR‐, metastasis‐free survival (MFS), and cancer‐specific survival (CSS) were estimated using the Kaplan‐Meier method.

**Results:**

Of 39 patients enrolled, 36 patients completed all study interventions. Eighteen (66.7%) patients had NCCN very high‐risk disease or clinical regional lymph nodes on imaging. Four patients (11.1%) achieved pCR, although two (5.6%) developed BCR. One patient (2.8%) had M1 and three (8.3%) had N1 disease on final pathology, and all four developed metastases. Eleven (30.6%) patients received salvage therapy, with all but one receiving ADT with radiation. Factors associated with BCR included biopsy ISUP grade and positive surgical margins, while NCCN risk group, biopsy ISUP grade, perineural invasion, and pathological stage were associated with metastases (*p* < 0.05). Median follow‐up was 7.3 (95% CI 6.3–8.3) years, and the 5‐year BCR‐free survival, MFS, and CSS were 64.1%, 84.6%, and 94.3%, respectively.

**Conclusions:**

Neoadjuvant enzalutamide and ADT was associated with favorable long‐term oncologic outcomes, supporting continued investigation in localized PCa.

**Abbreviations**
ADT: androgen deprivation therapy
ARSI: androgen receptor signaling inhibitor
BCR: biochemical recurrence
CI: confidence interval
CSS: cancer‐specific survival
GG: grade group
ISUP: International Society of Urological Pathology
MFS: metastasis‐free survival
mpMRI: multiparametric magnetic resonance imaging
NCCN: national comprehensive cancer network
PCa: prostate cancer
pCR: pathologic complete response
PSA: prostate‐specific antigen
RARP: robot‐assisted radical prostatectomy

## 1 Introduction

In 2025, it is estimated that prostate cancer (PCa) will be the second most common malignancy in men in the United States, contributing to nearly one‐third of all cancer diagnoses in men [1]. Despite attempts at definitive local therapy with surgery, approximately one‐third of patients are likely to experience biochemical recurrence (BCR) [2, 3]. These patients are at increased risk of metastatic progression and cancer‐specific mortality without salvage local therapy [4, 5]. Robot‐assisted radical prostatectomy (RARP) has become the prevailing surgical approach, though modern series still report a cancer‐specific mortality of 2%–10% for intermediate and high‐risk disease after surgery, despite 15.2%–29.9% of these patients receiving adjuvant therapy [6].

Neoadjuvant therapy, though widely adopted in other solid tumors, has historically demonstrated limited survival benefit in PCa. Prospective trials evaluating intense neoadjuvant androgen deprivation therapy (ADT) with androgen receptor signaling inhibitors (ARSIs) have reported encouraging pathologic response rates, with minimal residual disease or pathological complete response (pCR) observed in 20%–30% of patients [7, 8, 9, 10]. However, the clinical implications of these pathologic endpoints remain under investigation, and long‐term outcome analyses are ongoing. Moreover, predictive biomarkers of response, including PTEN loss, androgen and HER2 receptor expression, and DNA damage repair gene alterations, are emerging as critical factors to guide patient selection, particularly in intermediate‐risk disease, to direct novel therapeutics.

The present study builds on this growing body of evidence by evaluating long‐term outcome measures in a study of neoadjuvant enzalutamide plus ADT before RARP in men with newly diagnosed, localized, and clinically node‐positive PCa.

## 2 Materials and Methods

### 2.1 Study Design and Patient Selection

A prospective, open‐label feasibility clinical trial was performed at the National Cancer Institute (NCI) with accrual from June 3, 2015, to December 1, 2019. Follow‐up intervals were determined per protocol until the completion of the original study and then at the treating physician′s discretion. All patients provided informed consent and were co‐enrolled in an institutional review board‐approved protocol (NCT00050752). The relevant institutional review board approved the protocol and its amendments, and the study was conducted in accordance with the International Council for Harmonization Good Clinical Practice guidelines and the principles of the Declaration of Helsinki. The methodology, including the study design, patient selection, predefined outcomes, and adverse events, has been previously reported [11]. No formal sample size calculation was performed, given that the original trial was designed as a feasibility study, though the original target accrual was 55 patients.

In this study, men with newly diagnosed intermediate‐very high‐risk localized and clinically regional node‐positive PCa were treated for 6 months with complete intense androgen blockade with enzalutamide, plus ADT with goserelin, leuprolide, or degarelix (based on discretion of the treating physician), followed by RARP. Eligible patients had pathologically confirmed intermediate‐, high‐, very‐high‐risk localized, and clinically regional lymph node‐positive PCa based on the National Comprehensive Cancer Network (NCCN) with no prior history of treatment for PCa [12]. Patients were required to have an Eastern Cooperative Oncology Group performance status of 0–1 and baseline testosterone levels of ≥ 100 ng/dL. Patients with distant metastatic disease beyond N1 (regional) lymph nodes as detected on conventional imaging studies (CT, mpMRI, or bone scan), or who did not complete the study treatment, were excluded (*n* = 3). One patient died from a recreational drug overdose during the first month of treatment, while a second patient was unable to undergo RARP due to anesthesia concerns, and one patient progressed to metastatic disease on‐treatment [11].

### 2.2 Study Treatment

Patients received enzalutamide 160 mg administered orally daily for 28 days, for six cycles, plus ADT administered subcutaneously at Weeks 0 and 12 during treatment. This was followed by RARP and pelvic lymph node dissection (obturator and external iliac chains). The American Urologic Association (AUA) definition of BCR after prostatectomy was used, which is defined as the date of the first PSA ≥ 0.2 ng/mL followed by a confirmatory PSA ≥ 0.2 ng/mL [13]. Prostate biopsy and final pathology were reported using the International Society of Uropathology (ISUP) grade group (GG) system and by consensus from two unblinded expert genitourinary pathologists [14].

### 2.3 Data Collection

Clinicopathologic characteristics, patient demographics, clinical data, and therapeutic interventions were extracted from electronic medical records, and patients were censored at their last follow‐up. Prognostic stratification was performed on pre‐neoadjuvant therapy characteristics using NCCN risk groups [12].

### 2.4 Statistical Analysis

Continuous variables′ means ± standard deviations or medians and interquartile ranges [25th to 75th percentile] were calculated based on the data distribution. The comparison of numerical variables between groups was performed using the Student′s *t*‐test or Mann–Whitney *U*‐test as appropriate. Categorical variables were analyzed using a *χ* ^2^ or Fisher′s Exact test, as applicable. The Kaplan‐Meier method was used to estimate the BCR‐free survival, metastasis‐free survival (MFS), and cancer‐specific survival (CSS), though the median was not reached for each outcome. The median BCR‐free survival, MFS, CSS, and follow‐up were calculated. Additionally, survival tables were developed to assess the cumulative proportion of patients at the end of each interval. Missing data were handled using pairwise deletion, and a *p*‐value of < 0.05 was considered statistically significant. Statistical analysis was conducted using SPSS v.30 software. The International Committee of Medical Journal Editors (ICMJE) recommendations were followed in preparing this manuscript.

## 3 Results

### 3.1 Patient Demographics and Clinical Features

A total of 36 patients underwent neoadjuvant enzalutamide and ADT before RARP. The median age at surgery was 66.0 [59.1–70.8] years, and 25 (69.4%) were White. Patients had a median baseline PSA of 10.1 [6.0–22.8] ng/dL, PSA density of 0.26 [0.16–0.57], and a prostate volume of 41.0 [32.3–53.6] cc, with 30 (83.3%) of the patients having ≥ 1 PIRADS 5 lesion on multi‐parametric magnetic resonance imaging (mpMRI). Additionally, 15 (44.1%) patients had ISUP GG 5 adenocarcinoma on prostate biopsy, and half of the patients (50.0%) were NCCN very high‐risk pretreatment (Table 1).

**Table 1:** Baseline clinical, demographic, pathological, and oncological characteristics.

Variables		Patients (*n* = 36)
Age at RARP, median [IQR]		66.0 [59.1–70.8]
Race, *n* (%)		
White		25 (69.4)
Black		5 (13.9)
Other^a^		6 (16.7)
BMI, mean ± SD		28.2 ± 3.9
NCCN Risk Group, *n* (%)		
Favorable intermediate		1 (2.8)
Unfavorable intermediate		5 (13.9)
High risk		6 (16.7)
Very high risk		18 (50)
Regional node positive (clinical)		6 (16.7)
Baseline PSA, median [IQR]		10.1 [6.0–22.8]
Baseline PSAd, median [IQR]		0.26 [0.16–0.57]
mpMRI		
PIRADS score, *n* (%)		
3		1 (2.8)
4		5 (13.9)
5		30 (83.3)
cN+		6 (16.7)
Prostate Volume, median [IQR]		41.0 [32.3–53.5]
Biopsy ISUP Grade Group, *n* (%)		
2		2 (5.9)
3		6 (16.7)
4		13 (36.1)
5		15 (41.7)

Abbreviations: BMI, body mass index; IQR, interquartile range; NCCN, National Comprehensive Cancer Network; PI‐RADS, Prostate Imaging Reporting and Data System; PSA, prostate‐specific antigen; PSAd, PSA density; RARP, robot‐assisted radical prostatectomy; SD, standard deviation.

^a^ Asian, Hispanic, and multi‐racial.

### 3.2 Outcomes

On final pathology, the median prostate weight was 35.2 [28.2–46.4] g; 12 (33.3%) had extra‐prostatic extension, 5 (13.9%) had lymphovascular invasion, and 7 (19.4%) had seminal vesicle invasion. There were 7 (19.4%) positive surgical margins, 4 (11.1%) had positive lymph nodes, one of which was beyond the true pelvis (M1). A pCR was observed in 4 (11.1%) patients, while 28 (77.8%) had residual adenocarcinoma with observed treatment effect, and 4 (11.1%) of patients had residual adenocarcinoma with minimal treatment effect observed. During follow‐up, 35 (97.2%) patients had an undetectable nadir PSA, 14 (41.2%) developed BCR, 6 (16.7%) developed metastatic disease, and there were 2 (5.6%) PCa‐specific mortalities. Following BCR, one patient (2.8%) remains on surveillance, 11 (30.6%) received salvage therapy with radiation +/− ADT, and three patients (8.3%) developed metastatic disease before salvage therapy, two (5.6%) of which had a PCa‐specific mortality. The patient with M1 disease had evidence of progressive metastatic disease 1 month post‐surgery, with evidence of castration resistance, and was enrolled in a clinical trial for Durvalumab and Olaparib, followed by Pembrolizumab after disease progression, and subsequently, cancer‐specific mortality. The second patient had a detectable PSA 8 months postoperatively and had a subsequent 27‐fold increase in his PSA 3 months later, and he underwent combined ADT and docetaxel, and subsequently, cancer‐specific mortality. The third patient had a delayed presentation, and his PSA was 14.2 ng/µL with evidence of metastases, and he was given Docetaxel + Abiraterone with a sustained response. Of the remaining patients with metastases, one patient is pending treatment, two are on combination ADT + ARSi, and one patient received RT; however, their systemic therapy status is unknown. The median follow‐up was 7.3 [6.3–8.3] years, with a maximum follow‐up of 9.3 years (Table 2).

**Table 2:** Postoperative clinical, demographic, pathological, and oncological characteristics.

Variables		Patients (*n* = 36)
Prostate weight (g)		35.2 [28.2–46.4]
Pathological staging		*n* (% total)
ypT0		4 (11.1)
ypT2a		6 (16.7)
ypT2b		1 (2.8)
ypT2c		11 (30.6)
ypT3a		3 (8.3)
ypT3b		4 (11.1)
ypT3N1		3 (8.3)
ypT4N1M1		1 (2.8)
Positive margins		7 (19.4)
Lymphovascular invasion		5 (13.9)
Perineural invasion		18 (50.0)
Undetectable Nadir PSA		35 (97.2)
Last PSA		
Undetectable		26 (76.5)
0.01 to 0.2		3 (8.8)
≥ 0.2		5 (14.7)
Adjuvant therapy		0
BCR		14 (41.2)
Salvage		10 (30.6)
Type of salvage therapy		
RTx + ADT		10 (27.8)
RTx		1 (2.8)
Metastases^a^		7 (19.4)
Cancer‐specific survival		34 (94.4)
Median follow‐up in years [IQR], max		7.2 (6.3–8.3), 9.3

Abbreviations: ADT, androgen deprivation therapy; BCR, biochemical recurrence, NCCN® definition of 2 consecutive PSAs ≥ 0.2 after an undetectable nadir; PSA, prostate‐specific antigen; RTx, radiotherapy.

^a^ Treatment outlined in the manuscript.

Significantly different factors between those who did and did not have BCR included ISUP GG on pretreatment biopsy (*p* = 0.038), and positive surgical margins (*p* = 0.008), while, as expected, these patients were significantly more likely to develop metastases (*p* = 0.019). Two of the four (50%) patients with a pCR subsequently developed BCR, though neither developed metastatic disease following salvage therapy. All but one patient had high‐risk BCR (> 9 months PSA doubling time), with a median doubling time of 4.3 months [2.4–5.3], and this was not significantly different between those who developed metastases and those who did not (Table S1). All but one patient (83.3%) of the six patients who developed metastatic disease had ISUP GG 5 disease on prostate biopsy before initiation of neoadjuvant therapy. The ISUP GG on initial biopsy (*p* = 0.003), NCCN risk groups (*p* = 0.007), and final pathologic staging (*p* = 0.021) were all statistically different between those who developed metastases and those who did not (Table S2). All four patients who had positive lymph nodes identified on final pathology subsequently developed metastases.

**Figure 1:**
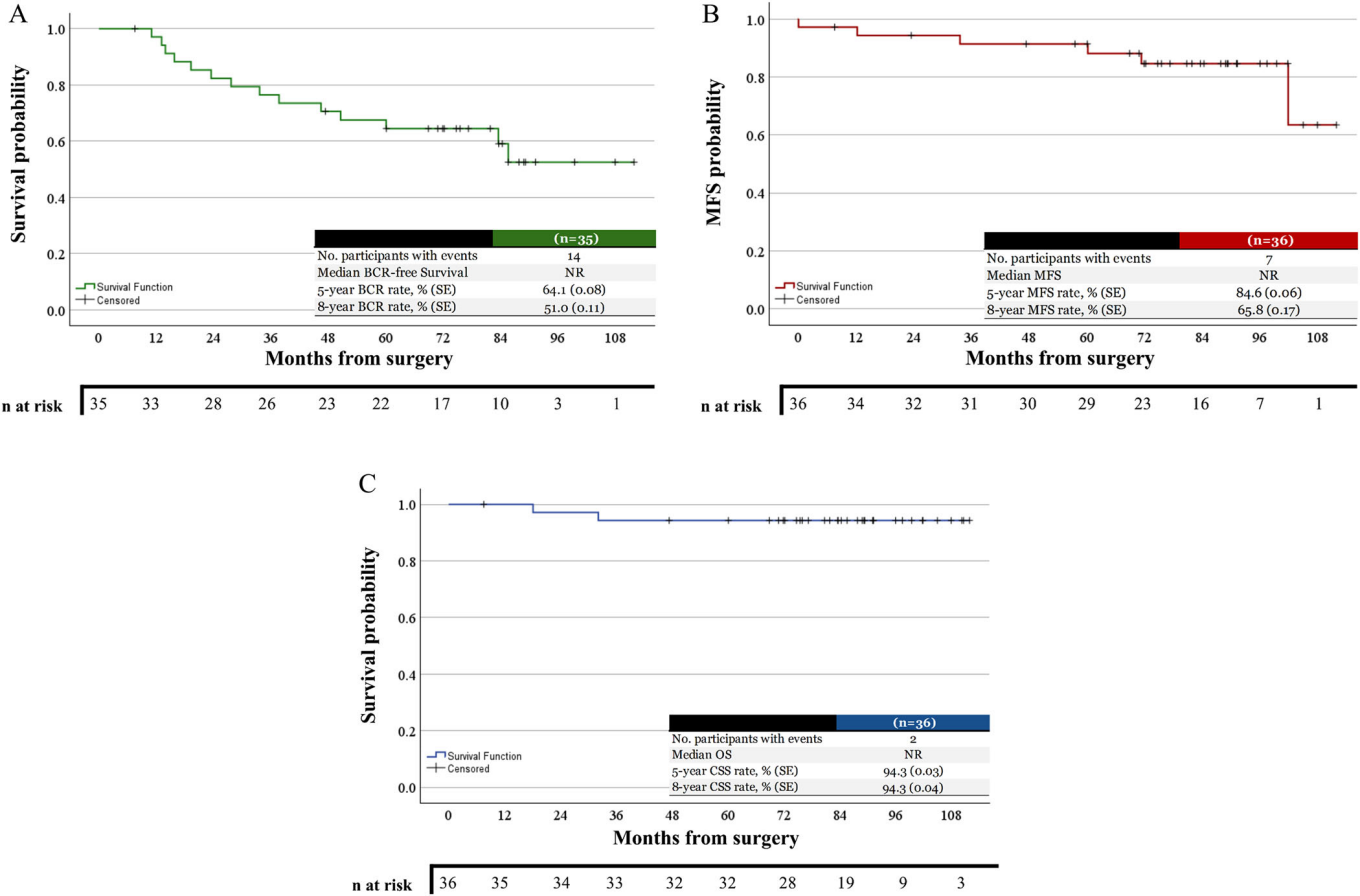
Kaplan–Meier curves showing (A) biochemical recurrence‐free (BCR) survival, (B) Metastasis‐free survival (MFS), and (C) cancer‐specific survival (CSS) following neoadjuvant intense androgen deprivation therapy and radical prostatectomy. NR, not reached. [Color figure can be viewed at wileyonlinelibrary.com]

The Kaplan‐Meier method could not estimate the median time to events (not reached); however, the BCR‐free survival rates at 5 and 8 years after RARP were 64.1% and 51.0%, respectively. The MFS rates at 5 and 8 years were 84.6% and 65.8%. At both 5 and 8 years, the OS and CSS were 94.3% (Figure 1). The calculated median BCR‐free survival was 71.9 months (IQR 33.6–85.6 months), MFS was 81.3 months (IQR 62.3–91.2 months), and the CSS was 84.9 months (IQR 72.8–97.1 months).

## 4 Discussion

In a secondary analysis of this open‐label feasibility trial, we evaluated the long‐term outcomes of neoadjuvant enzalutamide and ADT before RARP in men with localized NCCN intermediate‐, high‐, or very high‐risk PCa. In a population containing 50.0% NCCN very high‐risk and 16.7% patients with clinically positive regional lymph nodes, we observed a 5‐year BCR‐free survival of 64.1%, and a median time to development of BCR of 71.9 months. The pCR rate was limited, with four patients achieving pCR, which did not result in sustained BCR‐free survival. More than a third of patients underwent salvage therapy, while the 5‐year MFS and CSS were 84.6% and 94.3%, respectively, with a median follow‐up of 7.2 years.

Our findings show a durable response and BCR‐free survival following neoadjuvant enzalutamide and ADT, followed by RARP, extending prior work that primarily focused on early pathologic endpoints. In a large cohort of NCCN® high or very high‐risk patients, the median time to BCR was 10.8 and 14.3 months, respectively, which resulted in an observed cancer‐specific mortality (CSM) of 6% at a median follow‐up of less than 5 years [15]. Boorjian and colleagues have previously demonstrated that a shorter time to BCR is associated with increased CSM 10 years after RARP (9.9% CSM if BCR < 1.2 years vs. 4.7% CSM if BCR > 5.9 years), though this was not significant after multivariable adjustment [16]. Two of the four patients in our study who had a pCR developed BCR within 24 months of surgery, which contrasts the previously reported pooled analyses of neoadjuvant ADT + ARSI where there was an observed inverse association between the volume of residual disease and time to BCR [10]. These results still leave the long‐term benefits of neoadjuvant ADT + ARSI in PCa unanswered, while we await the results of significant phase III trials.

We observed a pCR in 11.1% and treatment effect in 77.8% of patients, confirming the incomplete response of localized PCa to intense androgen signaling inhibition. These rates are consistent with prior phase II studies, although we are the first to report the long‐term follow‐up [10]. Our study provides evidence that patients with pCR may go on to develop BCR, underscoring that pCR alone may not be sufficient as an endpoint for survival in neoadjuvant treatment studies in PCa, although none of these patients developed metastases. However, this observation is confounded by the absence of pretreatment molecular imaging, as some patients may have had more advanced disease than conventional imaging could detect. Additionally, possible mechanisms of resistance for AR‐directed therapy and other possible biomarkers need further evaluation in this population. Sowalsky and colleagues have described a possible mechanism for this resistance, whereby HER2 activity was positively associated with worse outcomes and opposed AR activity [17]. Therefore, investigating other neoadjuvant agents for high‐, very high‐risk, and regional node positive PCa is warranted.

Half of our cohort had NCCN very high‐risk, and 16% had regional node‐positive PCa before the initiation of neoadjuvant therapy, a population of patients with historically poor oncologic outcomes. In 2014, when the subgroup of NCCN patients with multiple high‐risk factors was first described, they reported a 10‐year MFS and CSS of 37% and 62%, respectively [18]. Though our median follow‐up is 7.2 years, and we included NCCN intermediate‐risk patients, we observed favorable 5‐ and 8‐year MFS and CSS. These findings are consistent with pooled analysis from previous phase II neoadjuvant studies, though longer follow‐up is required to determine the true benefit [19].

This secondary analysis of a feasibility study with a small sample size (*n* = 36) limits generalizability and precludes direct comparison to standard therapy. The heterogeneity in ADT agents used may introduce confounding, and three patients did not complete treatment and, therefore, were excluded from this analysis. Although long‐term follow‐up is reported, the number of metastatic and mortality events is low, limiting the interpretation of survival endpoints. The absence of a matched control arm limits the conclusions regarding the added benefit of neoadjuvant therapy beyond RARP alone in this patient population. Lastly, future studies need to incorporate serial molecular imaging.

## 5 Conclusion

The current study contributes to the growing literature on neoadjuvant enzalutamide plus ADT in localized intermediate‐, high‐, very high‐risk, and regional lymph node positive PCa by providing extended follow‐up data on oncologic outcomes. The observed rates of BCR‐free, MFS, and CSS support further investigation in larger randomized controlled trials with ARSIs and ADT.

## Study Need and Importance

After robot‐assisted radical prostatectomy (RARP), a high proportion of patients, especially those with very high‐risk disease, experience biochemical recurrence (BCR) with the potential for developing metastases and cancer‐specific mortality (CSM) To date, most neoadjuvant studies have focused on early time points, such as pathology complete response (pCR) or minimal residual disease (MRD) at the time of RARP, but the long‐term benefits of this approach remain unclear. This study aimed to see how well men did years after receiving intense neoadjuvant hormone therapy with enzalutamide plus androgen deprivation therapy (ADT).

## What We Found

In this study, 36 men, the majority with NCCN® very‐high and regional lymph node‐positive PCa, were treated with 6 months of hormone therapy before undergoing robotic prostate surgery and lymph node removal. After a median follow‐up of more than 7 years, 60% of men were BCR‐free, 83% had no evidence of metastases, and cancer‐specific survival was 94%.

## Limitations

This was a small feasibility study with only 36 patients and no comparison group, though historical cohorts of men with very‐high risk or regional lymph node positive have worse PCa outcomes. The use of different hormone therapy agents could also affect the results. Because there were few deaths and metastases, it′s hard to draw firm conclusions about survival benefits. Lastly, molecular imaging was not performed pre‐operatively for staging.

## Interpretation for Patient Care

This study suggests that adding enzalutamide and ADT before surgery may lead to long‐lasting control of PCa in men with very high‐risk and regional lymph node‐positive disease, though one‐third of patients still need salvage therapy, and 17% developed metastases. These findings support the idea of using intense hormone therapy before surgery in carefully selected patients. Though we await the results of large, randomized trials, patients can discuss the potential risks and benefits with their care team.

## Author Contributions

**Braden Millan:** conceptualization, methodology, investigation, formal analysis, writing – original draft, writing – review and editing. **Nikhil Pramod**, **Ruben Blachman‐Braun**, **Jaskirat Saini**, **Lauren Loebach**, **Milan Patel**, and **Sandeep Gurram:** data curation, writing – review and editing. **Baris Turkbey:** visualization, writing – review and editing. **Fatima Karzai:** conceptualization, supervision, resources, writing – review and editing. **Peter A. Pinto:** conceptualization, supervision, writing – review and editing.

## Ethics Statement

The patients presented in this study were co‐enrolled on the NCI/NIH IRB‐approved protocol ClinicalTrials.gov ID NCT02594202, and all patients provided informed consent to be part of this protocol. The neoadjuvant systemic therapy clinical trial was approved by the Institutional Review Board of the Center for Cancer Research, NCI (Bethesda, MD; ClinicalTrials.gov identifier: NCT02430480).

## Conflicts of Interest

The authors declare no conflicts of interest.

## Supporting information


**Supplementary Table 1:** Comparison of clinical, demographic, pathological, and oncological characteristics between patients that developed metastatic disease during follow‐up and those that did not.

**Supplementary Table 2:** Comparison of clinical, demographic, pathological, and oncological characteristics between patients who developed metastases and those who did not.

## Data Availability

Data are available upon request from the authors.
