# Supplementary material for: Long‐Term Follow‐Up of Neoadjuvant Enzalutamide Plus Androgen Deprivation Therapy in Localized Prostate Cancer: A Secondary Analysis of a Neoadjuvant Feasibility Trial
Source: Prostate. 2025 Nov 16;86(3):365–71. doi: 10.1002/pros.70093 (PMC12789907; doi:10.1002/pros.70093)
Supplement: Supplementary file 1 — Supplementary Table 1: Comparison of clinical, demographic, pathological, and oncological characteristics between patients that developed metastatic disease during follow‐up and those that did not. [file PROS-86-365-s001.docx]

| **Supplementary Table 1.** Comparison of clinical, demographic, pathological, and oncological characteristics between patients that developed metastatic disease during follow-up and those that did not. | | | |
| --- | --- | --- | --- |
| Variables | No BCR | BCR | p-value |
| n | 21 | 14 |  |
| Age at RARP, median [IQR] | 63.3 [58.8-71.4] | 67.7 [61.6 - 71.1] | 0.672 |
| NCCN ®, n (%) |  |  |  |
| Favorable Intermediate | 1 (4.8) | 0 |  |
| Unfavorable Intermediate | 4 (19.0) | 1 (7.1) |  |
| High risk | 4 (19.0) | 2 (14.3) |  |
| Very high risk | 9 (42.9) | 9 (64.3) |  |
| Regional | 3 (14.3) | 2 (14.3) | 0.189 |
| Baseline PSAd, median [IQR] | 0.21 [0.16-0.50] | 0.25 [0.14-1.40) | 0.194 |
| Baseline PSA, median [IQR] | 10.4 [6.0 - 22.8] | 10.2 [5.4-36.6] | 0.254 |
| PIRADS score, n (%) |  |  |  |
| 3 | 0 | 1 (7.1) |  |
| 4 | 4 (19.0) | 1 (7.1) |  |
| 5 | 17 (81.0) | 12 (85.7) | 0.895 |
| ISUP GG on Biopsy, n (%) |  |  |  |
| 2 | 1 (4.8) | 1 (7.1) |  |
| 3 | 6 (28.6) | 0 |  |
| 4 | 9 (42.9) | 4 (28.6) |  |
| 5 | 5 (23.8) | 9 (64.3) | **0.038** |
| Positive LN, n (%) | 0 | 3 (21.4) | 0.082 |
| ISUP GG Final Pathology, n (%) |  |  |  |
| Benign | 2 (9.5) | 2 (14.3) |  |
| 2 | 1 (4.8) | 0 |  |
| 4 | 0 | 1 (7.1) |  |
| 5 | 0 | 1 (7.1) |  |
| Treatment effect* | 18 (85.7) | 10 (71.4) | 0.67 |
| Positive margins, n (%) | 0 | 6 (42.9) | **0.008** |
| Lymphovascular invasion, n (%) | 1 (4.8) | 3 (21.4) | 0.194 |
| Perineural invasion, n (%) | 9 (42.9) | 8 (57.1) | 0.425 |
| Pathological Staging, n (%) |  |  |  |
| ypT0 | 2 (9.5) | 2 (14.3) |  |
| ypT2a | 4 (19.0) | 2 (14.3) |  |
| ypT2b | 0 | 1 (7.1) |  |
| ypT2c | 9 (42.9) | 2 (14.3) |  |
| ypT3a | 2 (9.5) | 1 (7.1) |  |
| ypT3b | 2 (9.5) | 2 (14.3) |  |
| ypTanyN1 | 0 | 3 (21.4) | 0.255 |
| PSA doubling time (mo), median [IQR] | - | 4.3 [2.4-5.3] | - |
| Most recent PSA, n (%) |  |  |  |
| Undetectable | 21 (100) | 10 (71.4) |  |
| 0.01 to 0.2 | 0 | 1 (7.1) |  |
| ≥ 0.2 | 0 | 3 (21.4) | 0.158 |
| Metastasis, n (%) | 0 | 5 (35.7) | **0.019** |
| Cancer-specific Survival, n (%) | 0 | 1 (7.1) | 0.336 |
| Median OS, years [IQR] | 6.8 [5.9-7.4] | 7.8 [6.9-8.6] | 0.090 |
| * Residual adenocarcinoma with treatment effect. |  |  |  |
