# Supplementary material for: Long‐Term Follow‐Up of Neoadjuvant Enzalutamide Plus Androgen Deprivation Therapy in Localized Prostate Cancer: A Secondary Analysis of a Neoadjuvant Feasibility Trial
Source: Prostate. 2025 Nov 16;86(3):365–71. doi: 10.1002/pros.70093 (PMC12789907; doi:10.1002/pros.70093)
Supplement: Supplementary file 2 — Supplementary Table 2: Comparison of clinical, demographic, pathological, and oncological characteristics between patients who developed metastases and those who did not. [file PROS-86-365-s002.docx]

| **Supplementary Table 2.** Comparison of clinical, demographic, pathological, and oncological characteristics between patients who developed metastases and those who did not. | | | |
| --- | --- | --- | --- |
| Variables | No metastatic disease | Metastatic disease | p-value |
| n | 29 | 7 |  |
| Age at RARP, median [IQR] | 63.8 [58.8-70.6] | 66.5 [61.5-72.4] | 0.262 |
| NCCN ®, n (%) |  |  |  |
| Favorable Intermediate | 1 (3.4) | 0 |  |
| Unfavorable Intermediate | 5 (17.2) | 0 |  |
| High risk | 6 (20.7) | 0 |  |
| Very high risk | 13 (44.8) | 5 (71.4) |  |
| Regional | 4 (13.8) | 2 (28.6) | **0.007** |
| Baseline PSAd, median [IQR] | 0.22 [0.16-0.50] | 0.41 [0.08-4.6] | 0.272 |
| Baseline PSA, median [IQR] | 9.6 [6.0-20.4] | 19.9 [5.53-272.1] | 0.254 |
| PIRADS score, n (%) |  |  |  |
| 3 | 0 | 1 (14.3) |  |
| 4 | 5 (17.2) | 0 |  |
| 5 | 25 (82.8) | 6 (85.7) | 0.712 |
| ISUP GG on Biopsy, n (%) |  |  |  |
| 2 | 2 (6.9) |  |  |
| 3 | 6 (20.7) |  |  |
| 4 | 12 (41.4) | 1 (14.3) |  |
| 5 | 9 (31.0) | 6 (85.7) | **<0.001** |
| ISUP GG Final Pathology, n (%) |  |  |  |
| Benign | 4 (13.8) | 0 |  |
| 2 | 1 (3.4) | 0 |  |
| 4 | 0 | 1 (14.3) |  |
| 5 | 1 (3.4) | 1 (16.7) |  |
| Treatment effect* | 23 (79.3) | 5 (71.4) | 0.264 |
| Positive margins, n (%) | 3 (10.3) | 4 (57.1) | 0.061 |
| Lymphovascular invasion, n (%) | 2 (6.9) | 3 (42.9) | 0.129 |
| Perineural invasion, n (%) | 12 (41.4) | 6 (85.7) | **0.024** |
| Pathological Staging , n (%) |  |  |  |
| ypT0 | 4 (13.8) | 0 |  |
| ypT2a | 8 (27.6) | 0 |  |
| ypT2b | 0 | 1 (16.7) |  |
| ypT2c | 10 (34.5) | 2 (28.6) |  |
| ypT3a | 3 (10.3) | 0 |  |
| ypT3b | 4 (13.8) | 0 |  |
| ypTanyN1 | 0 | 3 (42.9) |  |
| ypTanyN1M1 | 0 | 1 (14.3) | **0.021** |
| PSA persistence | 0 | 1 (14.3) | 0.356 |
| BCR | 7 (24.1) | 6 (85.7) | **<0.001** |
| PSA doubling time (mo), median [IQR] | 4.3 [2.5 – N.R.] | 3.0 [1.9 – 6.6] | 0.8 |
| Most recent PSA, n (%) |  |  |  |
| Undetectable | 28 (93.3) | 4 (66.7) |  |
| 0.01 to 0.2 | 0 | 0 |  |
| ≥ 0.2 | 2 (6.6) | 2 (33.3) | 0.571 |
| Salvage | 7 (24.1) | 4 (57.1) | 0.168 |
| Type of salvage Therapy |  |  |  |
| RTx + ADT | 6 (20.7) | 4 (57.1) |  |
| RTx | 1 (3.4) | 0 | 0.356 |
| Cancer-specific Survival, n (%) | 0 | 2 (28.6) | 0.175 |
| Median OS, years [IQR] | 7.1 [6.1-7.8] | 7.0 [2.7-9.2] | 0.677 |
| * Residual adenocarcinoma with treatment effect. | | |  |
